# Extreme medical misconduct: 49 UK-registered doctors involved in child pornography, dealt with by the General Medical Council 2012–2020

**DOI:** 10.1177/00258172231159765

**Published:** 2023-03-29

**Authors:** Hania Khalid, Timothy J David, Sarah Ellson

**Affiliations:** 1 Faculty of Biology, Medicine and Health, University of Manchester, Manchester, UK; 2 Fieldfisher, Manchester, UK

**Keywords:** Medical regulation, child pornography, medical misconduct, child abuse, General Medical Council, Medical Practitioners Tribunal Service

## Abstract

**Background:**

Between 1 June 2012 and 31 December 2020, there were 49 cases considered by the Medical Practitioners Tribunal Service where a doctor’s misconduct involved child pornography. The determinations concerning these cases provided the data for analysis.

**Findings:**

In 47/49 (96%) the regulatory outcome was erasure from the GMC’s Medical Register, ending the doctor’s career. 12 doctors had been imprisoned for 1 to 20 years, and 19 given suspended prison sentences. In 33/49 (67%) cases the indecent images of children included one or more video recordings. Some of these were of children (including very young infants) being raped, sometimes for prolonged periods, the video recordings sometimes indicating that the child could be seen to be in extreme pain.

**Interpretation:**

The high proportion of erasures reflected the gravity of these cases, coupled with the fundamental incompatibility of sexual misconduct involving children with a career in medicine.

## Introduction

Since 1858, the General Medical Council (GMC) has been responsible for regulating doctors in the United Kingdom. The GMC investigates complaints against doctors, and refers the most serious cases to a fitness to practise tribunal. Since 11 June 2012, with the aim of achieving better separation between the investigation and adjudication function, and to take responsibility for their daily management, these hearings have been managed by a separate body, the Medical Practitioner Tribunal Service (MPTS)^1^ which is accountable to the GMC. MPTS tribunals decide on any necessary sanctions, the most serious of which is the erasure of a doctor’s name from the Medical Register, which brings a doctor’s medical career to a halt. Detailed determinations, which are published on the MPTS website where they are available for 12 months, explain the decisions made.

The involvement of doctors in child pornography has only been noted in one small study, conducted between 2003 and 2005 in the UK and the USA, a total of 32 cases,^2^ and is a topic that (surprisingly) has not been studied in depth. There have been a number of published studies from the USA and Australia concerning the general topic of sexual misconduct by doctors,^3–7^ but most did not appear to involve children or child pornography. The present study differs from previous published studies of child pornography by focusing on the involvement of doctors, and by addressing the nature of the misconduct and the outcomes of both the regulatory and the criminal prosecution processes.

There have been widespread concerns regarding undue leniency of medical regulators when they deal with cases of sexual misconduct by doctors. In the UK these concerns came to a head in 1998 with a televised report of a series of women who were shocked to find that the doctor who had sexually abused them and been erased from the GMC Medical Register was restored to the Register and permitted to return to unrestricted clinical practice after only a short period.^8^ The response to this concern included a marked tightening of the GMC regulations concerning erasure from the Medical Register, which now require an absolute minimum of 5 years before a doctor can apply for restoration to the Register, with no guarantee of success.^9^ A study of 1039 USA physicians reported for sexual misconduct between 2003 and 2013 found that more than two-thirds of the doctors had not been disciplined by any state medical board,^10^ and a further study in the USA reported that a substantial proportion of physicians disciplined for sexual offences were allowed to either continue to practise or return to practice.^11^ Similar concerns were published regarding a failure of medical regulators in the USA to take steps to protect the public from 3,500 doctors involved in sexual misconduct in the period 1999 to 2016.^12^ A failure of regulators to act to protect the public from harm by offending doctors has also been reported in Australia.^13^ A contributory problem is that doctors in Australia who have had their licence to practise removed for serious sexual misconduct were in a number of cases able to return to practice through a process that often did not enter the public domain.^14^

## Methods

### The GMC investigation

If the police are aware that the perpetrator of a serious criminal offence is a doctor (a “notifiable profession”) they are likely to alert the GMC which will then launch its own investigation.

When investigating reports of criminal behaviour by a doctor, the GMC will take into account the following issues:

- the facts from the criminal court;
- the judge’s sentencing remarks;
- the nature of the sentence handed down and the person’s inclusion on the Sex Offenders’ Register;
- the severity of the offence;
- the degree of remorse shown;
- estimates of the likelihood of repeat offending;
- the effect the case has on public confidence in the medical profession.

### The MPTS tribunal process

The MPTS tribunal process follows a series of recognisable stages.

***Stage 1: The facts stage.*** The MPTS panel will decide that an allegation is proved if it is more likely than not to have happened – the civil standard of proof.

***Stage 2: Decision on impairment.*** The panel will consider, on the facts found proved, whether the facts indicate impairment of fitness to practise.

***Stage 3: Sanctions.*** The panel decides what sanction, if any, should be imposed, taking into account the GMC-MPTS Sanctions Guidance which outlines the factors to be considered when deciding upon a suitable sanction.^15^

The doctor, the GMC, and the Professional Standards Authority for Health and Social Care (PSA) all have the power to appeal against an MPTS decision. The outcomes recorded in this study were the final outcome of the case if an appeal had been held.

Criminal convictions may inherently establish the sexual misconduct on which the MPTS can base a decision about impairment. The criminal cases in the series reported here had all been considered by the MPTS after the criminal procedures had been completed, with one exception where the MPTS considered the case before the criminal proceedings had been completed.

### Inclusion criteria

The GMC/MPTS provided the published determinations for all 1948 UK-registered doctors whose case was considered by an MPTS medical practitioner tribunal and concluded between 11 June 2012 and 31 December 2020. The 49 cases that involved child pornography were identified by the authors and selected for this study.

### Variables recorded

Where the information was available in the determinations, the following variables were recorded in each case:

- the location at which the doctor obtained their primary medical qualification;
- details of the misconduct;
- in some cases the criminal charges specified whether the images were still or moving – the number of cases involving moving images was recorded;
- the outcome of the MPTS tribunal in terms of the sanction;
- sentences imposed by courts at criminal hearings.

### Terminology and relevant UK legislation concerning child pornography cases

The terminology used in criminal cases has evolved during the period of study (2012–2020). During the period of this study, a number of different classifications of degrees of seriousness of indecent images were in use. A complicating feature was that in a number of the cases included in this study the criminal offences occurred in different parts of the UK, the British Isles, or overseas, where different laws and/or terminology were in use. As a result we have not attempted to classify the seriousness of the images.

The term “extreme pornographic images” is applied to offences such as a person performing sex with a live animal, or an act which involves sexual interference with a corpse, or (for example) extreme pornographic images which portrayed, in an explicit and realistic way, an act which resulted, or was likely to result, in serious injury to a person’s private parts and which was grossly offensive, disgusting or otherwise of an obscene character.

### Additional sentences for criminal offences

In addition to imprisonment, additional sentences, such as the making of a sexual harm prevention order (SHPO), or requiring the offender to sign on the Sex Offenders Register (SOR), were noted. Community orders were noted. Possible sentences not recorded here included ordering participation in an internet sex offender treatment programme, a rehabilitation activity requirement, and making an order for the offender to be deprived of rights in respect of computers and external devices used to store indecent images.

### Implications of the sex offender register and sexual harm prevention orders

The implications of the GMC/MPTS sanctions guidance and relevant case law^16^ is that a doctor should not be permitted to resume unrestricted practice until satisfactory completion of a criminal sentence.

## Results

All 49 doctors involved in child pornography had received criminal convictions, which were as follows:

- Community orders or probation in 18/49 (36.7%) cases, ranging from 12 months to 3 years.
- Prison sentences requiring imprisonment, 12/49 (24.5%), ranging from 1 to 20 years or over.
- Suspended prison sentences, 19/49 (38.8%), ranging from 4 months to 2 years.
- Sex offender orders were made in 43/49 (87.8%) cases, ranging from 2 years to an indefinite duration.
- Sexual harm prevention orders were made in 35/49 (71.4%) cases, ranging from 2 years to an indefinite duration.

In 12/49 (24.5%) cases there was reference to the finding that a doctor had been in possession of so-called “extreme” pornographic images.

### Location of primary medical qualification (PMQ)

The data for the 49 doctors and for all UK registered doctors 2012–2020 was as follows:

The location of the PMQ was the UK in 35/49 (71.4%) of the doctors studied and 1,610,831/2,568,093 (62.7%) of all UK registered doctors.

The location of the PMQ was Europe in 6/49 doctors studied (12.2%) and 274,885/2,568,093 (10.7%) of all UK registered doctors.

The location of the PMQ was the rest of the world in 8/49 (16.3%) of the doctors studied and 682,377/2,568,093 (26.6%) of all UK registered doctors.

### The number of indecent images of children involved

Information on the total number of images was available in 41/49 (83.7%) cases, and was as follows:

- up to 999: 20;
- 1000–99,999: 17;
- 100,000 or more: 4.

### Video recordings

In 33/43 (76.7%) cases the indecent images of children included one or more video recordings. The accompanying text in some cases provided descriptions indicating that the videos were of children being raped, sometimes for prolonged periods, the video recordings sometimes indicating that the child (including very young infants) could be seen to be in extreme pain. The presence or absence of video recordings, as opposed to still images, was not systematically recorded in the MPTS determinations, and it is possible the data reported here has under-estimated the number of cases involving moving images.

### Additional criminal charges and additional allegations made by the GMC

In addition to charges relating to indecent images, there were 5 cases that involved convictions for voyeurism. There was one doctor who had been found to have conducted a number of unnecessary internal examinations. Two cases involved the doctor grooming a child and inciting a child to have sex. In one case the doctor was found by the MPTS to have failed to notify the GMC that he faced criminal charges, and that he failed to notify the GMC of his criminal convictions. One doctor was convicted of four counts of possession of a class B drug and one count of “theft by employee”.

### MPTS tribunal outcomes

In 2/49 (4.1%) cases the outcome was suspension for 12 months, followed by a review hearing, resulting in both doctors being permitted to resume clinical practice without restrictions. In 47/49 (95.9%) the outcome was erasure. This included one case in which the MPTS panel directed suspension for 12 months, which was appealed by the PSA, with the result that an order was made for erasure.

## Discussion

### Methodological limitations

This study only refers to cases of child pornography that were detected and reported to the GMC. We have no information about the number of undetected or unreported cases. Another limitation is that this study provides no information that could shed light on the cause of the misconduct; the MPTS tribunal process is not focused on finding the root causes for human behaviour. In addition, all matters relating to a doctor’s health are regarded as confidential and redacted from the determinations, so it is not possible to glean any information about any underlying health (e.g. mental health) issues that may have caused or contributed to the actions of the doctor.

MPTS determinations were not created with the aim of providing material for research. Whilst there was a certain basic structure and layout that applied to all determinations, there was no information about the age of the doctors, and there was a variable level of information concerning the specialty of practice. The retrospective nature of the study coupled with the variation in approach in time and the different legislation in different geographical locations meant that there was no systematic approach to the recording of details of the crimes and the sentences, and the level of detail varied from case to case.

### Protecting the public from doctors involved in child pornography

To protect the public the first lines of defence are the police, the criminal justice system, a medical regulatory system, and in the UK, the Barring List system. The UK Disclosure and Barring Service has two barred list offence databases on which someone’s name can be placed, the Children’s and Adult’s. If one’s name is on a Barred List, it is against the law to seek to be engaged in a role that includes a Regulated Activity^17^ with the concerned group. A person’s name can be reported to the Disclosure and Barring Service by members of the public, employers and other bodies, but automatic barring can occur when someone has been cautioned or convicted of a relevant offence, or has been issued with an SHPO. Examples of offences that can lead to being added to a Barring List include rape, murder, sexual assault, cruelty to persons under 16, sexual intercourse with someone under 16, and possession or distribution of indecent images of children. Most of the sexual offences referred to in this study would result in automatic barring.

### The controversial nature of the term child pornography

The term “pornography” is generally taken to refer to still photographs, video recordings, other images or writings whose purpose is to elicit sexual arousal. Where one or more children are depicted, the term “child pornography” has been in widespread use. Drawbacks to the term are (i) it may be felt to de-emphasise the harm done to the victims who have been exploited in order to produce images; (ii) some victims and survivors feel the term legitimises the activity; (iii) it fails to reflect the lack of consent from the victims of the abuse; (iv) it might be seen to treat the material as a legitimate subgenre of adult pornography. However it is difficult to avoid the term given its prominence in international law and human rights instruments, and this use does not appear to be changing.^18^ While the term “child pornography” has been used in this paper to describe this category of offence, in part because it is a term that was until recently commonly used by the MPTS, the formal description of the relevant criminal charges referred to making (including downloading), possessing, and/or sharing “indecent images or pseudo-photographs”.

### Place of medical qualification

It is known that doctors who trained overseas are more likely to be referred to the GMC and to receive sanctions by an MPTS tribunal.^19^ The numbers in our study are insufficient for detailed statistical analysis, but the relative dearth of overseas graduates among the child pornography users was consistent with the finding in a review of 26 published studies examining the characteristics of online offenders that online (as compared with offline) sex offenders are more likely to be Caucasian.^20^
